# GDF-15 as a Weight Watcher for Diabetic and Non-Diabetic People Treated With Metformin

**DOI:** 10.3389/fendo.2020.581839

**Published:** 2020-11-18

**Authors:** Jing Ouyang, Stéphane Isnard, John Lin, Brandon Fombuena, Xiaorong Peng, Yaokai Chen, Jean-Pierre Routy

**Affiliations:** ^1^ Chongqing Public Health Medical Center, Chongqing, China; ^2^ Infectious Diseases and Immunity in Global Health Program, Research Institute, McGill University Health Centre, Montréal, QC, Canada; ^3^ Chronic Viral Illness Service, McGill University Health Centre, Montréal, QC, Canada; ^4^ CIHR Canadian HIV Trials Network, Vancouver, BC, Canada; ^5^ Department of Microbiology and Immunology, McGill University, Montréal, QC, Canada; ^6^ State Key Laboratory for Diagnosis and Treatment of Infectious Diseases, National Clinical Research Center for Infectious Diseases, Collaborative Innovation Center for Diagnosis and Treatment of Infectious Diseases, The First Affiliated Hospital, College of Medicine, Zhejiang University, Hangzhou, China; ^7^ Division of Hematology, McGill University Health Centre, Montréal, QC, Canada

**Keywords:** metformin, body weight, obesity, GDF-15, GFRAL, diabetes

## Abstract

Weight gain and obesity are global health concerns contributing to morbidity with increased risks of cardiovascular disease, diabetes, liver steatohepatitis and cancer. Pharmacological therapies or bariatric surgery are often required for those who fail to adhere to diet and lifestyle modifications. Metformin, a widely used antidiabetic agent, seems to have a health benefit beyond its anti-hyperglycemic properties, with few side effects. Emerging evidence shows weight loss to be associated with metformin in both diabetic and non-diabetic individuals. Recently, the growth differentiation factor 15 (GDF-15), a member of the transforming growth factor beta superfamily, has been identified as a key mediator of metformin-induced weight loss. Metformin increases the secretion of GDF-15, which binds exclusively to glial cell-derived neurotrophic factor family receptor alpha-like (GFRAL). This gut-brain cytokine works as a prominent player in reducing food intake and body weight in health and disease, like anorexia nervosa and cancer. Herein, we critically review advances in the understanding of the weight-reducing effects of metformin *via* the GDF-15 pathway.

## Introduction

Weight gain and obesity represent the second most common causes of preventable mortalities worldwide, and promote the development of cardiovascular disease, hypertension, stroke, dyslipidemia, metabolic syndrome, liver steatohepatitis, and cancer (1, 2). Diet and lifestyle modifications are the first line interventions for the management of body weight, however long-term adherence remains difficult. As such, pharmacological interventions are needed. However, due to the cost and the risk of side effects, few drugs are currently available.

Isolated from French lilac in 1920s, metformin (dimethylbiguanide) has been widely used as a first-line treatment for type 2 diabetes mellitus (DM2), owing to its excellent tolerability, safety profile, and lack of hypoglycemic effect (3–6). Aside from its anti-hyperglycemic effects, metformin has an additional benefit for conditions like polycystic ovary syndrome, atherosclerosis, cancer, coronavirus disease 2019 (COVID-19), and obesity (7–14). Metformin was also reported to extend lifespan in some animal models, acting as a diet mimetic agent (15–17). In contrast to other antidiabetic drugs including insulin, metformin can lead independently to glycemic control and weight loss by decreasing food intake in both diabetic and non-diabetic individuals (18–20). Thus, these observations have driven metformin’s emergence as a research priority to counteract diseases associated with obesity and aging, evocating an immune-metabolic effect.

Bodyweight is usually maintained through central nervous system (CNS) circuitry integrating peripheral metabolic feedback signals of either energy surplus or deficit (21, 22). Studies have shown converging evidence that growth differentiation factor 15 (GDF-15) mediates metformin’s effect on the gut-nervous system axis to decrease body weight (23–26). GDF-15, a member of the transforming growth factor beta (TGF-β) superfamily, has many names indicating its multiple functions, including macrophage inhibitory cytokine (MIC)-1, non-steroidal anti-inflammatory drug-inducible gene (NAG)-1, placental TGF-β (PTGF), prostate-derived factor (PDF), and placental bone morphogenetic protein (PLAB) (27). GDF-15 is a stress-induced protein, produced by a variety of cells under stress conditions including tissue injury, anoxia, and inflammation (28). Elevated circulating GDF-15 levels have been associated with more severe disease or higher mortality in people with DM2, insulin resistance, hemodialysis, cachexia, cardiovascular diseases, chronic obstructive pulmonary disease (COPD), venous thromboembolism (VTE), cancer, or obesity (28–34). Husebø et al. reported that high levels of GDF15 were independently associated with a higher rate of COPD exacerbations, and impaired respiratory function (34). From a prospective cohort of 27,158 adults followed over 13 years, among 12 tested biomarkers, GDF-15 and D-dimer were independently associated with the occurrence of VTE in multivariable analyses. Importantly, the association between GDF-15 levels and VTE was independent of D-dimer and von Willebrand factor, which are well-established biomarkers for thrombosis (35). Therefore, the elevation of GDF-15 levels during those conditions are believed to be a compensatory mechanism, as numerous evidences proposed GDF-15 as a biomarker rather than an inducer of these diseases. GDF-15 has also been shown to inhibit apoptosis and inflammation, and protects the heart from injury (36–38). In addition to these functions, GDF-15 and its tissue-specific brainstem receptor, the glial cell-derived neurotrophic factor (GDNF) family receptor alpha-like (GFRAL), have emerged as regulators of energy balance and body weight (39–41). Higher circulating GDF-15 levels have been proven to be significantly associated with weight loss in cancer patients (42, 43). To review the influence of the GDF-15 pathway on weight loss induced by metformin, we searched for the keywords “GDF-15”, “metformin”, “weight”, “diabetes drugs” alone, or in combination in the public databases of PubMed, Google Scholar, and ClinicalTrials.gov. As the relationship between GDF-15 and anti-diabetic drugs is new, we were able to discuss all English publications. In this review, we critically discuss advances in the understanding of the weight-reducing effects of metformin through modulation of GDF-15 levels in diabetic and non-diabetic individuals.

### Metformin Decreases Body Weight in Diabetic and Non-Diabetic Individuals

Dysglycemia is strongly associated with the development of overweight status or obesity, as most DM2 patients are overweight or obese (44–46). For diabetic patients, managing overweight or obesity is a crucial facet of diabetes management. However, the majority of antidiabetic agents, including insulin, thiazolidinediones, (TZDs) and insulin secretagogues, lead to body weight gain while controlling glycemia (47–49). Conversely, the first-line antidiabetic agent, metformin, has been shown to be able to decrease body weight (18, 19, 49–52). Kahn et al. (49) reported in a large randomized clinical trial (RCT) involving 4360 DM2 patients that participants lost a mean of 2.9 kg with metformin over a period of 5 years, while rosiglitazone and glyburide both induced weight gains of 4.8 and 1.6 kg, respectively. The Diabetes Prevention Program Research Group reported that metformin users had significantly reduced body weight and waist circumference compared with placebo in a 2-year RCT followed by a 8-year open-label extension. The magnitude of weight loss during the 2-year double-blind period was directly related to drug adherence, indicating a potential dose-effect (19). In 2016, a meta-analysis by Maruthur et al. showed that metformin decreased body weight more than dipeptidyl peptidase-4 (DPP-4) inhibitors which were expected to decrease body weight (18). Due to the effects of weight loss, metformin was recommended for obesity management in patients with evidence of prediabetes or insulin intolerance by AACE/ACE guidelines (53).

Aside from diabetic patients, metformin was also associated with a decrease of bodyweight in non-diabetic subjects (20, 54–57). Ejtahed et al. (20) demonstrated in a RCT that metformin induced significant weight loss compared with placebo, and this effect was associated with gut microbiota alteration in non-diabetic obese women. Furthermore, metformin has been used in obese children to promote weight loss in absence of DM2 (58–61). For adults and children with schizophrenia, metformin has been used to manage weight gain associated with anti-psychotic drugs, reducing risks of metabolic syndrome, diabetes and cardiovascular disorders (62–66). Moreover, obesity and insulin resistance are associated with pathogenesis of polycystic ovarian syndrome (PCOS), a condition characterized by a reduced frequency of ovulation, infertility, and hyperandrogenism in premenopausal women (67, 68). Metformin’s effect on weight in women with PCOS is not as well defined, depending on the population and study design (69–74). However, a meta-analysis showed that metformin contributed to a decrease of body mass index (BMI) and waist to hip ratio (WHR) in 11 and 7 RCTs of PCOS women respectively, compared to placebo (75). Finally, a 12-week metformin treatment decreased the weight of non-diabetic people living with HIV under antiretroviral therapy (25).

Metformin use has been associated with weight loss and it is noteworthy that metformin-associated weight loss was of a lesser extent in non-diabetic people. As such obese people may benefit from bariatric surgery more readily than from metformin (76). Although most studies have confirmed that metformin could decrease body weight in diabetic and non-diabetic subjects, the mechanism still remains unclear. Aside from the mechanisms summarized in two reviews (77, 78), recent study findings indicate that GDF-15 plays an independent role in body weight change in people taking metformin.

### Metformin Induces GDF-15 *Via* ATF4 and CHOP

Converging findings showed that metformin induced *gdf-15* gene expression and elevated the circulating GDF-15 level in animal and human models (**Table 1**). Metformin-induced expression was notably detected in gut and kidney epithelial cells (23). Metformin and other biguanides such as phenformin were shown to induce GDF-15 expression in murine and human hepatocytes (23), while the direct effect of other anti-diabetes drugs has not been studied yet. *In vitro*, 1mM of metformin upregulated GDF-15 gene expression in breast cancer cells to 26-fold compared with control (79). Higher concentrations of metformin (10-100 mM) in mesenchymal stem cells (MSCs) increased GDF-15 expression in a dose-dependent manner under normoglycemic conditions. Interestingly, this effect was hindered in hyperglycemic conditions (80). *In vivo*, Gerstein et al. assayed 237 biomarkers in baseline serum from 8,401 participants with dysglycemia of whom 2,317 received metformin and found that GDF-15 was linked to metformin treatment, in a dose dependent manner (1 per mg of metformin treatment led to 8.7 pg/ml of GDF-15 increased in plasma). Moreover, Coll et al. (23) reported in two independent RCT that metformin reduced food intake and lowered body weight in association with increasing levels of GDF-15. However, the same group showed that metformin retained its ability to lower circulating glucose and fasting insulin levels in GDF-15 knock-out mice. These findings suggest that GDF-15 mediates the beneficial effects of metformin on energy balance and weight loss, independently of insulin pathways. However, based on the negative effect of weight on insulin sensitivity, it was speculated that GDF-15-dependent weight loss contributed to enhance insulin sensitivity. We conducted a prospective study to examine the effect of metformin on body weight in non-diabetic, non-obese people living with HIV and receiving effective antiretroviral therapy (ART). We showed that metformin, independent of its glucose-lowering effect, increased plasma levels of GDF-15 and decreased weight, and its effects vanished upon discontinuation, establishing a direct cause-effect relationship between GDF-15 plasma level change and weight change during and after metformin discontinuation (25).

**Table 1:** Reports of metformin’s effects on GDF-15 in different models.

Study, year	Models	Number	Dose of metformin	Change of GDF-15 by metformin
**Animal studies**				
Day et al., 2019 (24)	Mice fed with chow diet and high fat diet	n = 6-7 per group	A single oral gavage of metformin (250 mg/ kg) or an equal volume of saline	Metformin significantly increased serum GDF-15 in both chow diet and high fat diet groups
Coll et al., 2020 (23)	Obese mice	Three groups: Vehicle, Metformin (300 mg/kg) Metformin (600 mg/kg), n = 7 per group,	Single oral dose of 300 or 600 mg/kg	300 mg/kg of metformin increased GDF-15 levels for at least 8 h. 600 mg/kg of metformin resulted in a six-fold increase in serum GDF-15 levels at 4 h and 8 h after the dose.
**Cellular studies**				
Williams et al., 2013 (79)	MDA-MB-468 breast cancer cells	n = 3 per group	Cells were cultured for 48 h in the absence or presence of 1 mM metformin	GDF-15 gene expression was increased 25.61 fold in metformin group compared with control.
Zafarvahedian et al., 2017 (80)	Mesenchymal stem cells (MSCs)	n = 3 per group	MSCs were treated with 10, 50, and 100 mM metformin for 17 h	GDF-15 production was increased in a dose dependent manner. GDF-15 levels increased by dose up to 2-fold control group levels at 100 mM.
Day et al., 2019 (24)	Primary mouse hepatocytes	(1) *n* = 4 per group (2) *n* = 3-6 per group	(1) Cells were treated with 0.5 mM metformin for 24 h (2) 0-1,000 μM for 24 h	(1)**** Metformin treatment significantly increased GDF-15 mRNA levels. (2)**** Metformin increased GDF-15 release in a dose-dependent manner
**Clinical trials**				
Gerstein et al., 2017 (81)	People with diabetes, impaired glucose tolerance, or impaired fasting glucose levels	8,401 participants (2,317 receiving metformin)	Various doses	Mean GDF-15 concentrations rose with metformin dose. GDF-15 was strongly linked to metformin, such that the odds of metformin use per standard deviation value increase in level varied from 3.73 (95% CI 3.40, 4.09) to 3.94 (95% CI 3.59, 4.33) depending on included variables.
Natali et al., 2018 (82)	Diabetic patients	644 (Metformin) vs 299 (Non-metformin)	Not mentioned	Metformin treatment was associated with a 40% rise in GDF-15 level, which was independent of the other major factors.
Coll et al., 2020-2 (23)	Overweight individuals	9 (placebo-controlled, double-blind crossover design)	Week 1: 500 mg twice daily; week 2: 1,000 mg twice daily.	After two weeks of metformin treatment, there was an increase of about 2.5-fold in mean circulating GDF-15.
Coll et al., 2020-3 (23)	Overweight or obese non-diabetic participants	86 (Metformin) vs 85 (placebo)	850 mg daily for 18 months	Metformin treatment was associated with significantly increased levels of circulating GDF-15 at all three time points (6, 12 and 18 months)
Isnard et al., 2020 (25)	Non-diabetic People living with HIV	Metformin	850 mg twice daily for 12 weeks.	Metformin treatment was associated with significantly increased levels of circulating GDF-15 at 12 weeks. Plasma GDF-15 levels went back to baseline levels 12 weeks after metformin discontinuation.

Although the mechanism responsible for metformin-induced GDF-15 expression is not yet deciphered, several pathways have been described to induce GDF-15 expression. In diabetic patients, hyperglycemia causes a stress condition leading to reactive oxygen species (ROS) overproduction, which further induces cellular apoptosis, cellular injury and cell death by inhibiting the PI3K/AKT/eNOS/NO pathway and activating the NF-κB/JNK/caspase-3 pathway (83–85). *In vitro*, Li et al. revealed that high glucose could induce GDF-15 expression and secretion in cultured human umbilical vein endothelial cells in a ROS- and p53-dependent manner (86). The transcriptional factor p53 regulates GDF-15 expression and was shown to link GDF-15 with obesity and insulin resistance (87). Obesity promotes p53 activation in adipose tissue and leads to increased production of proinflammatory cytokines and increased insulin resistance. When p53 was inhibited by RNA silencing, the effect of GDF-15 induction by high glucose vanished (86). In humans, high levels of plasma GDF-15 have been associated with type 2 diabetes and cardiovascular events (88–90). However, GDF-15 was shown to protect human islet cells from apoptosis and was suggested to have a protective role in diabetic mice (91). Metformin is also suggested to prevent cardiovascular diseases and to have anti-aging effects (15, 92–94), although these results need to be confirmed (95). As such, the clinical implication of metformin-induced GDF-15 increase will have to be assessed in future RCTs.

Aside from p53, several other factors have been implicated in the transcriptional regulation of GDF-15, including p63, Sp1, early growth response-1 (EGR-1), activating the integrated stress response transcription factor 4 (ATF4), C/EBP homologous protein (CHOP), and SMAD2/3 (96–103). Patel et al. reported that GDF-15 levels increased following sustained high-fat feeding or dietary amino acid imbalance in mice, and that GDF-15 expression is regulated by the integrated stress response, in which key transcriptional regulators like ATF4 and CHOP are involved (102, 104). Induced GDF-15 expression by the stressor tunicamycin was abolished in ATF4 knockout mouse embryonic fibroblasts and significantly reduced in CHOP-knockdown cells (102). Similarly, Chung et al. (100) showed that induction of GDF-15 upon mitochondrial unfolded protein response (UPR^mt^) activation was CHOP-dependent. Interestingly, metformin was reported to increase the expression of ATF4 and CHOP, further stimulating the secretion of GDF-15 in mouse and human hepatocytes (24). Therefore, current evidence indicates that metformin increases GDF-15 gene expression and stimulate GDF-15 secretion by direct induction of integrated stress response regulators ATF4 and CHOP.

### GDF-15 and Weight Loss: Evidencesand Mechanism

A direct association between weight and GDF-15 was studied in animal and humans. Altered GDF-15 levels in comparison to matched lean controls have been frequently reported in obese mice, rats, and humans (32, 105). In addition, elevated GDF-15 expression and circulating levels correlate with further weight loss, reduce food intake and appetite (42, 43, 106). Patients with metastatic lung cancer who reported >5% weight loss were found to exhibit a twofold increase in GDF-15 plasma levels compared to those without weight loss (42). Tsai et al. (107) reported that GDF-15 gene knockout mice (*Gdf-15* ^(-/-)^) weighed more and had increased adiposity, which was associated with increased food intake, and that infusion of human recombinant GDF-15 was sufficient to raise serum levels in *Gdf-15* ^(-/-)^ mice to within the normal human range, reducing body weight and food intake. On the contrary, overexpressing GDF-15 led to decreased body weight, fat mass and food intake, improving the glucose tolerance in mouse models (83, 108–110). GDF-15 has thus become an attractive target for reducing obesity. In line with these studies, exogenous administration of recombinant murine or human GDF-15 induced weight loss in different animal models (26, 32, 40, 41, 107). Xiong et al. (32) showed that Fc fusion GDF-15 molecules with extended half-life and increased efficacy in obese mice, rats, and cynomolgus monkeys was able to delay gastric emptying, change food preference, and activate area postrema neurons, confirming a role for GDF-15 in the gut-brain axis responsible for the regulation of body energy intake. Moreover, pharmacological recombinant human GDF-15 administration to mice can trigger conditioned-taste aversion, suggesting that GDF-15 may induce an aversive response to nutritional stimulation and may be associated with nausea in pregnancy (102). These results were further confirmed by Coll et al. as metformin did not induce weight loss when administered to gdf-15 knock out mice (23).

**Figure 1:**
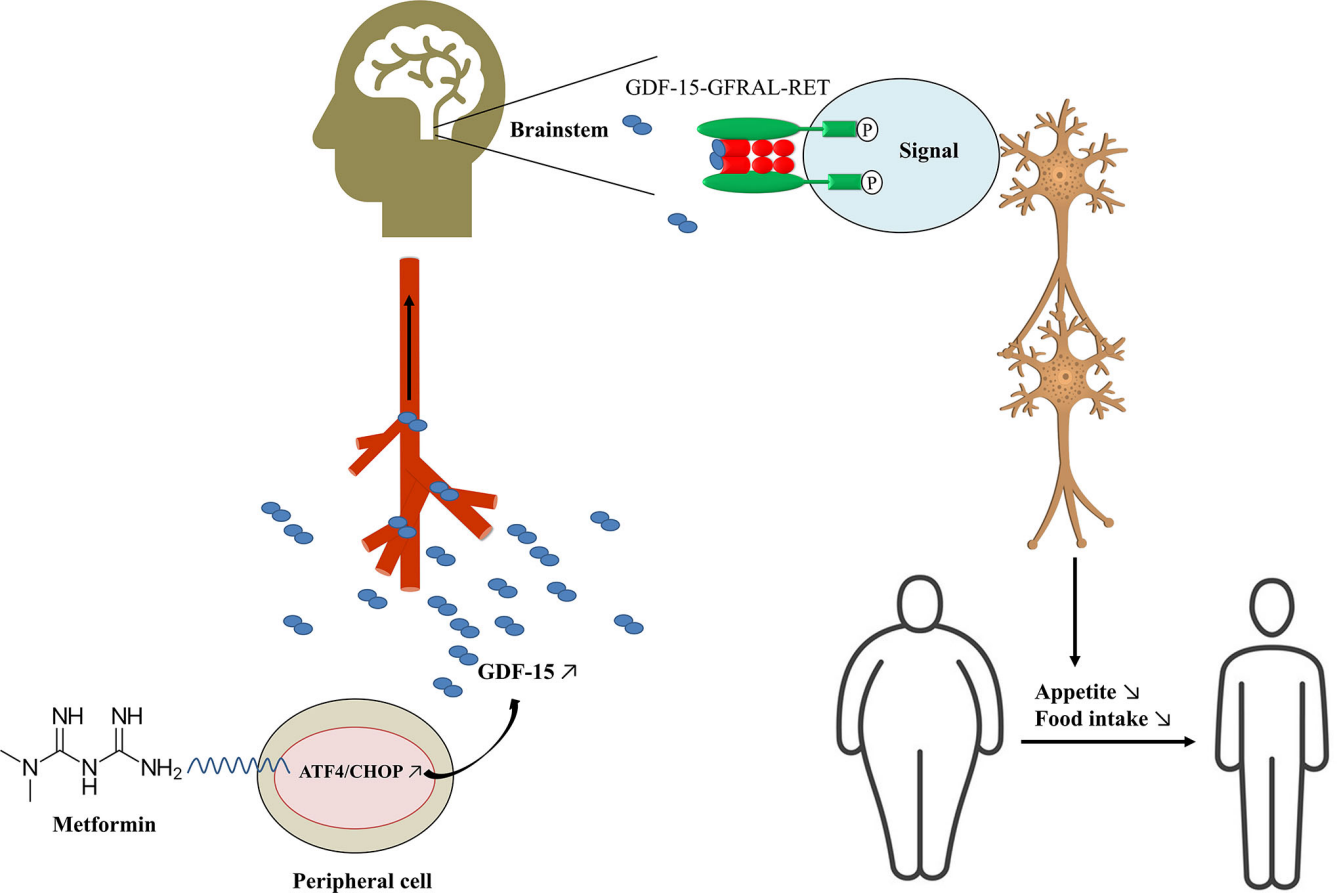
Potential mechanism of metformin decreasing body weight. GDF-15, growth differentiation factor 15; GFRAL, glial cell-derived neurotrophic factor (GDNF) family receptor alpha-like (GFRAL); ATF4, activating transcription factor 4; CHOP, C/EBP homologous protein.

Mechanistically, the weight-related effect of GDF-15 is dependent on its receptor GFRAL and coreceptor tyrosine kinase RET (**Figure 1**). Unlike *Gdf-15* which is expressed in diverse tissues, including kidney, liver, gut, muscle, adipose, and placenta, GFRAL expression is limited to the brainstem and *Gfral* mRNA is highly expressed in the area postrema of mouse, rat, monkey and human (39–41). GFRAL was previously considered as an orphan receptor with no endogenous ligand (111, 112). Recently, GDF-15 was validated as the only GFRAL ligand with a high-affinity. Flow cytometry analyses showed that GFRAL solely bound to GDF-15, but not to GDNF and its homologs neurturin, artemin, and persephin (26). Interestingly, GDF-15 also exclusively binds to GFRAL, and not to any other TGF-β receptors nor to other members of the GDNF family of receptors (39–41). In the brain, activation of the GFRAL receptor leads to a complex activation of a neuronal network involving the nucleus of the solitary tract, the hypothalamus, and the central amygdala, reducing food intake and appetite (**Figure 1**) (96). *Gfral* knockout mice are hyperphagic under stressed conditions and are resistant to chemotherapy-induced anorexia and body weight loss. Moreover, the effect of GDF-15 on body weight and food intake reduction in wild-type mice was completely lost in *Gfral* knockout mice (39). GFRAL antibody blocked GDF-15-induced body weight and food-intake suppression in rats (39). Additionally, GDF-15-induced cell signaling requires the interaction of GFRAL with the coreceptor RET (39). GDF-15 forms a complex with GFRAL and RET on the cell surface, then triggers an intracellular signaling cascade through the extracellular signal-related kinase (ERK) pathway (40, 113). Blocking RET by inhibitor or mRNA depletion could also prevent GDF-15-mediated signaling in neuroblastoma cells (41).

## Conclusion

Metformin has emerged as an effective weight-reducing medication in different animal and human models by increasing GDF-15 levels, which works as a “weight watcher” to maintain homeostasis. Metformin induces the expression of integrated stress response regulators ATF4 and CHOP, which stimulates the secretion of circulating GDF-15, which then binds to its exclusive receptor GFRAL and coreceptor RET in the brainstem. Intracellular signaling of the GDF-15/GFRAL/RET leads to feelings of satiety and control of appetite, resulting in decreased body weight (**Figure 1**). However, more evidence is needed to verify the role of GDF-15 as a biomarker of metformin’s weight-reducing effects in diabetic and non-diabetic individuals. In the future, metformin dose and duration should be well-defined, and use GDF-15 as a biomarker of metformin-induced weight loss will have to be confirmed. Moreover, the clinical implication and use of GDF-15 as a biomarker should be studied in large RCTs.

## Author Contributions

JO and SI wrote the first draft of the manuscript. JL, BF, and XP provided critical revision of the manuscript. YC and J-PR conceived and designed the manuscript. All authors contributed to the article and approved the submitted version.

## Funding

Our research is funded by the Fonds de la Recherche Québec-Santé (FRQ-S): Réseau SIDA/Maladies infectieuses and Thérapie cellulaire, the Canadian Institutes of Health Research (CIHR; grants MOP 103230 and PTJ 166049), the Vaccines & Immunotherapies Core of the CIHR Canadian HIV Trials Network (CTN; CTN PT027), the Canadian Foundation for AIDS Research (CANFAR; grant 02-512), and CIHR-funded Canadian HIV Cure Enterprise (CanCURE) Team Grant HB2-164064.

## Conflict of Interest

The authors declare that the research was conducted in the absence of any commercial or financial relationships that could be construed as a potential conflict of interest.
